# Study of the Herbicidal Potential and Infestation Mechanism of *Fusarium oxysporum* JZ-5 on Six Broadleaved Weeds

**DOI:** 10.3390/microorganisms13071541

**Published:** 2025-06-30

**Authors:** Suifang Zhang, Haixia Zhu, Yongqiang Ma, Liang Cheng

**Affiliations:** Academy of Agriculture and Forestry Sciences, Qinghai University, Xining 810016, China

**Keywords:** weeds, herbicidal activity, crop safety, ultrastructure, fungal biocontrol

## Abstract

Weeds compete with crops for resources, posing multiple negative impacts for agricultural production systems and triggering degradation of ecosystem services (e.g., alterations in the soil microbial community structure). Under the guidance of green plant protection, the development of efficient biocontrol strains with environmentally friendly characteristics has become a crucial research direction for sustainable agriculture. This study aimed to develop a fungal bioherbicide by isolating and purifying a pathogenic fungal strain (JZ-5) from infected redroot pigweed (*Amaranthus retroflexus* L.). The strain exhibited pathogenicity rates ranging from 23.46% to 86.25% against six weed species, with the most pronounced control efficacy observed against henbit deadnettle (*Lamium amplexicaule* L.), achieving a pathogenicity rate of 86.25%. Through comprehensive characterization of cultural features, morphological observations, and molecular biological identification, the strain was taxonomically classified as *Fusarium oxysporum*. Scanning electron microscopy revealed that seven days post-inoculation, *F. oxysporum* JZ-5 formed dense mycelial networks on the leaf surfaces of cluster mallow (*Malva verticillata* L.), causing severe tissue damage. Safety assessments demonstrated that the spore suspension (10^4^ spores/mL) had no adverse effects on three crops: hulless barley (*Hordeum vulgare* var. *coeleste* L.), wheat (*Triticum aestivum* L.), and potato (*Solanum tuberosum* L.). These findings suggest that *F. oxysporum* strain JZ-5 warrants further investigation as a potential bioherbicide for controlling three problematic weed species—*Chenopodium album* L. (common lambsquarters), *Elsholtzia densa* Benth. (dense-flowered elsholtzia), and *Lamium amplexicaule* L. (henbit deadnettle)—in cultivated fields of hulless barley (*Hordeum vulgare* var. *coeleste* L.), wheat (*Triticum aestivum* L.), and potato (*Solanum tuberosum* L.). This discovery provides valuable fungal resources for ecologically sustainable weed management strategies, contributing significantly to the advancement of sustainable agricultural practices.

## 1. Introduction

The Qinghai–Tibetan Plateau, serving as the headwaters of the Yangtze, Yellow, and Lancang Rivers, possesses critical strategic importance for ecological conservation. However, this region’s distinctive extreme environmental conditions—including high altitude, low temperatures, intense radiation, and aridity [1]—result in an exceptionally fragile ecosystem with significantly reduced environmental carrying capacity compared to other regions [2]. Within this ecologically sensitive zone, weed communities exhibit complex structural composition, comprising perennial grasses (e.g., *Avena fatua*), annual herbs (e.g., *Chenopodium album* L.), broadleaf weeds (e.g., *Polygonum lapathifolium* L.), and invasive species (e.g., *Amaranthus retroflexus* L.), forming a diverse weed spectrum. According to the Weed Science Society of America database (Home—Weed Science Society of America), globally, there are 1118 documented weed species, classified into 177 families and 2847 genera [3]. These weeds compete with crops for essential resources including light, soil nutrients, growing space, carbon dioxide, and water, making them the primary biological factor responsible for crop yield reduction [4], with annual yield losses of approximately 30% in major crops worldwide [5].

Currently, chemical herbicides remain the predominant approach for weed control, yet their excessive application poses significant challenges to sustainable agricultural development [6]. Research indicates that only 10–30% of applied chemical herbicides are effectively adsorbed by target plants or soil particles, with the remainder entering surface runoff and groundwater systems through precipitation or irrigation [7]. This phenomenon not only contributes to water contamination, but may also induce phytotoxicity in non-target plants. These issues are particularly pronounced in the ecologically vulnerable Qinghai–Tibetan Plateau region, where the fragile ecosystem renders it more susceptible to cascading environmental consequences from herbicide use, including ecological pollution and the development of herbicide-resistant weed populations. Consequently, the exploration of environmentally benign weed-management technologies has become an urgent priority for agricultural development in this region.

From an academic perspective, the utilization of bioherbicides containing fungal active ingredients or natural fungal metabolites represents a promising alternative for reducing dependence on chemical products. Microbial-derived herbicides generally fall into two categories: (1) microbial inoculants employing live microorganisms (including spores, hyphae, and other viable components) that infect target weeds, causing disease or direct mortality; and (2) microbial metabolite-based herbicides, which are formulated from toxins or antibiotics with weed-suppressive properties obtained through microbial fermentation processes. Notably, fungal pesticides utilizing conidia as their primary active components accounted for 10% of the global biopesticide market in 2016 [8]. *Fusarium*, one of the most extensive fungal genera, is well-documented for causing various crop diseases including root rot, stem blight, and head blight. Emerging evidence suggests its promising potential as a bioherbicidal agent. Necajeva et al. [9] demonstrated that treatment of barnyardgrass (*Echinochloa crusgalli*) seeds with *Fusarium culmorum* spore suspensions (2.5 × 10^5^ spores/mL) resulted in 73.3% suppression of seed germination after 42 days. Similarly, Motlagh [10] reported that foliar application of *Fusarium equiseti* spores (1.0 × 10^5^ spores/mL) at the 2–3 leaf stage of early watergrass (*Echinochloa oryzicola*) induced severe leaf-spot symptoms, ultimately causing plant mortality. Furthermore, studies have identified specific *Fusarium* strains, such as *F. avenaceum* [11] and *F. culmorum* [12], capable of effectively colonizing and degrading wild oat (*Avena fatua*) caryopses.

However, the potential of *F. oxysporum* as a bioherbicide, particularly in the Qinghai Plateau region of China, remains unexplored. Against this background, this study aims to isolate and characterize naturally occurring fungal pathogens from diseased weeds in Qinghai, China, and evaluate their potential for weed management applications.

## 2. Materials and Methods

### 2.1. Survey Locations and Sampling

This study was conducted in farmland located in Jianzha County (35°56′12″ N, 102°01′85″ E, elevation: 3500 m), Xining City, Qinghai Province, to investigate weed infestation. The survey covered cultivated fields of wheat (*Triticum aestivum* L.), hulless barley (*Hordeum vulgare* L.), faba bean (*Vicia faba* L.), rapeseed (*Brassica napus* L.), pea (*Pisum sativum* L.), and potato (*Solanum tuberosum* L.).

Weed specimens exhibiting typical disease symptoms, including redroot pigweed (*Amaranthus retroflexus* L.), wild barley (*Hordeum vulgare* L.), and dockleaf smartweed (*Polygonum lapathifolium* L.), were collected. The samples were aseptically cut into 1 cm × 1 cm segments, placed in sterile bags, and transported to the laboratory for fungal isolation.

### 2.2. Isolation of the Pathogen

The experiment was conducted at the Qinghai Provincial Key Laboratory of Integrated Pest Management in Agriculture (36°73′07″ N, 101°75′96″ E, altitude 2263 m). Following the isolation method described by Ma Y [13], leaf samples were first rinsed under running water and air-dried on sterile filter paper. Leaf tissues (1 cm^2^) were excised from the margins of necrotic or chlorotic lesions, then surface-sterilized sequentially with 75% ethanol for 1 min and 1% sodium hypochlorite for 2 min, followed by rinsing with sterile distilled water for 2 min. Under aseptic conditions in a laminar flow hood, the sterilized tissue segments were transferred onto potato dextrose agar (PDA) plates, supplemented with ampicillin (1 µL/mL). The plates were incubated at 25 °C under a 12 h light/12 h dark photoperiod for 7 days. After repeated purification to obtain single colonies, isolated strains were transferred to PDA slants and stored at 4 °C for subsequent studies.

### 2.3. In Vitro Pathogenicity Assay of Strain JZ-5 on Leaf Segments of Four Weed Species

After culturing the pathogenic strain on PDA plates for 7 days, mycelial plugs (4 mm in diameter) were taken from the colony margins. Healthy leaves of four weed species—*Malva verticillata* L., *Polygonum lapathifolium* L., *Polygonum aviculare* L., and *Chenopodium album* L.—at the 4–5 leaf stage, were collected from the experimental fields of the Qinghai Academy of Agricultural and Forestry Sciences. Under sterile conditions in an SW-CJ-1D (Suzhou Purification Equipment Co., Ltd., Suzhou, China) laminar flow hood, the leaves were surface-sterilized with 1% sodium hypochlorite for 3 min, followed by three rinses with sterile distilled water. Pathogenicity testing was conducted using an agar plug inoculation method, where a 6 mm mycelial plug was placed at the center of each leaf. Control leaves were inoculated with sterile PDA plugs. The inoculated leaves were placed in Petri dishes (Φ = 90 mm) lined with moist filter paper, with three replicates per treatment and three independent experimental repeats. The plates were incubated in a growth chamber at 25 ± 1 °C under a 12 h light/12 h dark photoperiod. Disease severity was assessed daily, according to a 0–4 scale (modified from Reference [14]): 0 (no lesions), 1 (visible lesions), 2 (lesions covering 1/3–2/3 of the leaf area), 3 (necrosis exceeding 2/3 of the leaf), and 4 (complete leaf wilting and necrosis).

### 2.4. Pathogenicity Assessment of Strain JZ-5 on Six Weed Species

The fungal colonies cultured on PDA plates for 7 days were carefully scraped and suspended in 10 mL sterile distilled water. The resulting suspension was filtered through four layers of sterile gauze to remove hyphal fragments, followed by preparation of a spore suspension containing 0.01% Tween 80 as a surfactant. Preliminary experiments demonstrated that the optimal infection concentration was 10^6^ spores/mL. Concentrations below this threshold consistently resulted in infection failure, while higher concentrations led to inconsistent infection outcomes. Therefore, the stock inoculum was precisely adjusted to 10^6^ spores/mL, using a hemocytometer for subsequent experiments.

The weed seeds used in the experiment were collected from mature, dried plants in the field in 2022. The seeds were sown in 15 cm diameter plastic pots filled with a sterilized substrate mixture (leaf mold:garden soil:perlite:plant ash = 2:2:1:1, *v*/*v*). When the plants reached the 6–8 leaf stage under greenhouse cultivation, a spore suspension (10^6^ spores/mL) was applied using a sterilized spray bottle for whole-plant inoculation, with a controlled application volume of 30 mL per pot. A sterile water treatment served as the control. The greenhouse conditions were maintained at 25 °C with >80% relative humidity and a 12 h photoperiod. Disease development was assessed 7 days post-inoculation by calculating the disease incidence and disease index. Disease severity was rated daily, according to the established scale [14]: Grade 0 (no lesions); Grade 1 (leaf lesions visible); Grade 2 (lesions covering 1/3–2/3 of leaf area); Grade 3 (over 2/3 of leaf necrotic); and Grade 4 (complete leaf wilting and yellowing). Observations of lesion progression were recorded daily, throughout the experimental period.

$$Disease\;incidence\;rate\;(\%)=\frac{number\;of\;leaves\;affected}{total\;number\;of\;leaves\;surveyed}\times 100$$

$$Disease\;index=\frac{number\;of\;diseased\;leaves\times number\;of\;corresponding\;grades\;}{total\;number\;of\;leaves\;surveyed\times number\;of\;highest\;grades}\times 100$$

### 2.5. Safety Evaluation of Strain JZ-5 on Six Crop Species

A systematic host range investigation was conducted, targeting six agronomically important plant species across three botanical families in Qinghai Province, including hulless barley (*Hordeum vulgare* var. *coeleste* L.) at tillering stage, wheat (*Triticum aestivum* L.) at jointing stage, potato (*Solanum tuberosum* L.) at seedling stage, rapeseed (*Brassica napus* L.) at early growth phase, pea (*Pisum sativum* L.) at vine elongation, and faba bean (*Vicia faba* L.) at seedling establishment. All test plants were cultivated in 15 cm diameter pots containing sterilized growth substrate until reaching the 4–5 leaf stage, at which point they were inoculated using the standardized protocol described previously. Control groups received 0.1% Tween-80 aqueous solution. Post inoculation, plants were maintained in controlled-environment chambers at 25 ± 1 °C, with >80% relative humidity under a 12 h photoperiod. The experimental design incorporated three biological replicates per treatment with six independently repeated trials. Disease severity classification was established based on Disease Index (DI) values according to Reference [15], with minor modifications, categorizing pathogenic effects into four distinct grades: non-susceptible (NS, 0 ≤ DI < 5), low susceptibility (LS, 5 ≤ DI < 10), moderately susceptible (MS, 10 ≤ DI < 50), and highly susceptible (HS, DI ≥ 50). This standardized classification system facilitated quantitative evaluation of host–pathogen interactions across various crop developmental stages.

### 2.6. Molecular Identification and Characterization of Target Strain

#### 2.6.1. Identification Based on Morphological Characteristics

For microscopic examination, fungal isolates were cultured on Potato Dextrose Agar (PDA) plates under controlled conditions: incubation at room temperature (25 ± 1 °C) with a 12 h light/dark cycle for 7–15 days. Following isolation and purification, the strains were centrally inoculated on fresh PDA plates and allowed to grow for 7 days under the same conditions. Colony morphology was periodically assessed, including edge characteristics, elevation, presence of exudates, pigment deposition, and color variations on both the obverse and reverse sides of the culture. Fungal structures were then analyzed using a Nikon Eclipse E200 LED (Nikon Instruments Co., Ltd., Shanghai, China) optical microscope coupled with a Canon EOS 700D digital camera (Canon China Co., Ltd., Beijing, China) system to document hyphal types, reproductive morphologies, diameter, coloration, and other defining features. Microscopic observations of hyphae and spore morphology, combined with macroscopic colony traits, were cross-referenced with the *Fungal Identification Manual* [16] for preliminary taxonomic classification.

#### 2.6.2. Molecular Identification

The molecular identification of fungal isolates was performed by extracting total genomic DNA using a fungal genomic DNA extraction kit. PCR amplification was conducted with universal primers ITS1 (CCGTGTGTGCGG) and ITS4 (TCTCGCTTATTGTGGC), synthesized by Sangon Biotech (Shanghai, China). The 50 μL PCR reaction system consisted of 25 μL 2× PCR Master Mix, 2.0 μL each of forward and reverse primers (10 μmol/L), 1.0 μL DNA template (50 ng/μL), and ddH_2_O, to adjust the final volume to 50 μL. The thermal cycling conditions were as follows: initial denaturation at 95 °C for 5 min; followed by 35 cycles of 94 °C for 30 s, 65 °C for 30 s, and 72 °C for 90 s; with a final extension at 72 °C for 10 min. The amplified products were electrophoresed on a 2.0% agarose gel and subsequently sent to Sangon Biotech for purification and sequencing. The obtained sequences were aligned with 21 high-homology sequences and 3 outgroup strain sequences. Multiple sequence alignment was performed using the ClustalW algorithm in MEGA 11.0 software, followed by splicing of high-quality sequences. A phylogenetic tree was constructed using the Maximum Composite Likelihood method as the evolutionary model, with branch reliability assessed through 1000 bootstrap replications. The construction criteria prioritized DNA sequences, employed the Maximum Composite Likelihood method for tree building, and validated branch confidence through repeated sampling.

### 2.7. Ation of Strain JZ-5 for Electron Microscopy Observation on Malva verticillata L. (Cluster Mallow)

The purified strain JZ-5 was cultured on PDA plates at 25 °C for 5 d on treated weed leaves, and uninoculated PDA was used as a control. Samples were taken daily from 0–7 d and stored frozen at −20 °C in a freezer. The operational procedures were carried out at 4 °C throughout. The samples were rinsed with 0.1 mol/L of pH = 7.2 phosphate buffer with 15~20 min intervals between each change, for 1 h. After fixation with 4% glutaraldehyde for 2 h, the fixative was aspirated out, and the samples were rinsed with 0.1 mol/L of pH = 7.2 phosphate buffer with 15~20 min intervals between each change, for 1 h. After rinsing, the samples were dehydrated, and the commonly used dehydrating agent was ethanol. The buffer was aspirated from the vial, and ethanol was added for step-by-step gradient dehydration, at concentrations of 30–50–70–80–90–100% ethanol (2 times). The samples remained in the ethanol solution for 20 min at each step. When the subsequent experiments could not be completed on the same day as the other operational steps, the samples were stored in the 70% dehydrating agent. The ethanol was aspirated, and 1:1 mixture of isoamyl acetate and ethanol was added. The sample was soaked for 10~20 min and shaken appropriately, then the mixture was aspirated and pure isoamyl acetate was used to extract the sample for 10~20 min, with appropriate shaking. Coating and gold spraying with ion sputtering equipment, the vacuum was released, and the sample was placed under the scanning electron microscope (SEM) for observation and photography.

### 2.8. Data Processing

The data were processed and analyzed using Excel 2016 and IBM SPSS Statistics 25.0 software. Sequence analysis was performed using MEGA 11. Experimental data are presented as mean ± standard error (SE). Statistical significance of differences among mean values was determined by Duncan’s new multiple range test at a significance level of *p* ≤ 0.05. The raw data were subjected to normality testing (Shapiro–Wilk test) and homogeneity of variance testing (Levene’s test), with results indicating that all significance levels exceeded 0.05. Consequently, the data satisfied the normality and homogeneity of variance assumptions for parametric testing (ANOVA), thereby eliminating the need for data transformation.

## 3. Results

### 3.1. Pathogenicity Assay of Strain JZ-5 Spore Suspension on Detached Leaves of Four Weed Species

**Figure 1:**
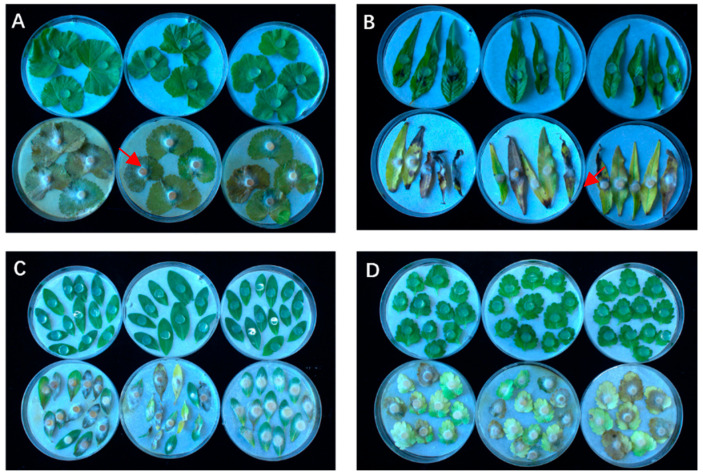
Pathogenicity of strain JZ-5 on isolated leaves of four weed species, the red arrows indicate hyphae. **Note**: (**A**) *Malva verticillata* L.; (**B**) *Polygonum lapathifolium* L.; (**C**) Polygonum aviculare L.; (**D**) *Lamium amplexicaule* L. The diameter of the Petri dish is 9 mm.

The fungal strain JZ-5 was inoculated onto target weed species using mycelial plug. Seven days post inoculation, disease progression was assessed, with results presented in Figure 1 and Table 1. *Malva verticillata* L. (Cluster mallow): leaves exhibited chlorosis and softening, accompanied by extensive mycelial growth on the surface. The total leaf area affected was 11.38 cm^2^, with a disease incidence area of 11.08 cm^2^. *Polygonum lapathifolium* L. (Pale smartweed): brown necrotic lesions developed around the inoculation sites, along with small brown spots and marginal curling. The total leaf area was 7.84 cm^2^, with a disease incidence area of 7.02 cm^2^. *Polygonum aviculare* L. (Prostrate knotweed): leaves showed chlorotic yellowing and were partially covered by mycelia. The total leaf area was 1.79 cm^2^, with a disease incidence area of 1.05 cm^2^. *Lamium amplexicaule* L. (Henbit deadnettle): leaves turned completely yellow-brown, with dense mycelial coverage. The total leaf area was 3.16 cm^2^, with a disease incidence area of 2.81 cm^2^. The following weeds exhibited severe infection: Pale Persicaria (*Polygonum lapathifolium* L.) and Henbit *Lamium amplexicaule* L. (Henbit), both with a disease severity rating of 5. In contrast, Common Knotgrass *Polygonum aviculare* L. (Common Knotgrass) showed moderate infection, with a disease severity rating of 2.

### 3.2. Pathogenicity Assay of Strain JZ-5 Spore Suspension on Six Potcultured Weed Species

**Figure 2:**
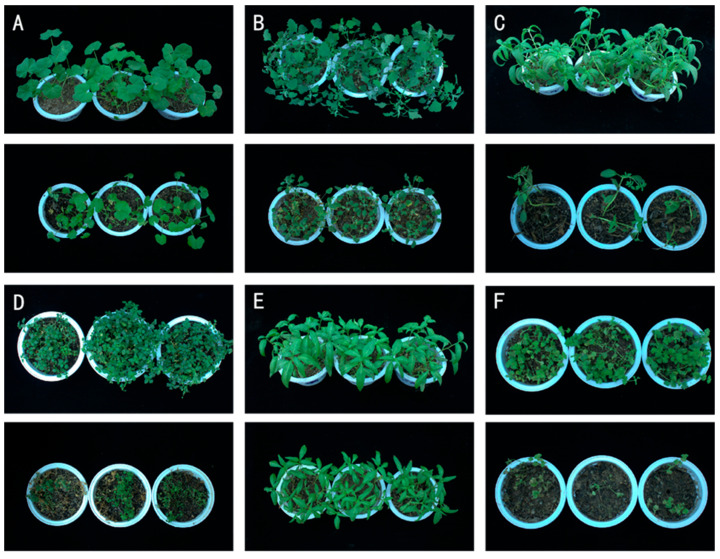
Pathogenicity of strain JZ-5 on six weed species in pot test. **Note**: (**A**) *Malva verticillata* L. (top is control and bottom is treatment for each weed, with the same below); (**B**) *Chenopodium album* L.; (**C**) *Elsholtzia densa Benth*.; (**D**) *Stellaria* media (L.) *Vill*.; (**E**) *Polygonum lapathifolium* L.; (**F**) *Lamium amplexicaule* L. The diameter of the Petri dish is 9 mm.

Seven days after inoculation with strain JZ-5 spore suspension, disease progression was assessed on test weeds (Figure 2 and Table 2). *Malva verticillata* L. (Cluster mallow) exhibited stunted growth accompanied by leaf chlorosis and irregular scattered lesions, with an incidence rate of 33.61% and disease index of 8.36%. *Chenopodium album* L. (Lamb’s quarters) displayed pronounced symptoms, with over 75% of leaves developing necrotic lesions. Chlorosis initiated at leaf margins and progressively expanded toward the center, ultimately leading to necrosis and significant growth inhibition (incidence: 76.83%; disease index: 38.36%). *Elsholtzia densa Benth*. (Dense-flowered elsholtzia) showed particularly effective pathogenic effects, with characteristic symptoms including dark-brown necrotic scorching along leaf margins, resembling fire damage. Severe cases exhibited stem wilting and necrosis (incidence: 81.71%; disease index: 61.26%). *Stellaria media* (L.) *Vill*. (Common chickweed) demonstrated extensive leaf yellowing and desiccation, followed by growth arrest and eventual plant death (incidence: 75.17%; disease index: 62.60%). *Polygonum lapathifolium* L. (Pale smartweed) showed minimal pathogenic effects, presenting only slight leaf curling and size reduction, without mortality, though growth was significantly suppressed (incidence: 23.46%; disease index: 5.83%). *Lamium amplexicaule* L. (Henbit deadnettle) exhibited the most severe infection among tested weeds, with initial symptoms appearing as chlorotic white spots along leaf margins that progressed to leaf curling, desiccation, and necrosis (incidence: 86.25%; disease index: 64.63%). Among the tested species, *Chenopodium album* L., *Elsholtzia densa Benth*., *Stellaria media* (L.) *Vill*., and *Lamium amplexicaule* L. showed severe disease symptoms with significant pathogenic effects, all reaching disease severity level 3. In contrast, *Malva verticillata* L. and *Polygonum lapathifolium* L. showed markedly milder infection, with disease severity levels of 2 and 1, respectively.

### 3.3. Safety Evaluation of Strain JZ-5 Spore Suspension on Four Crop Species

**Figure 3:**
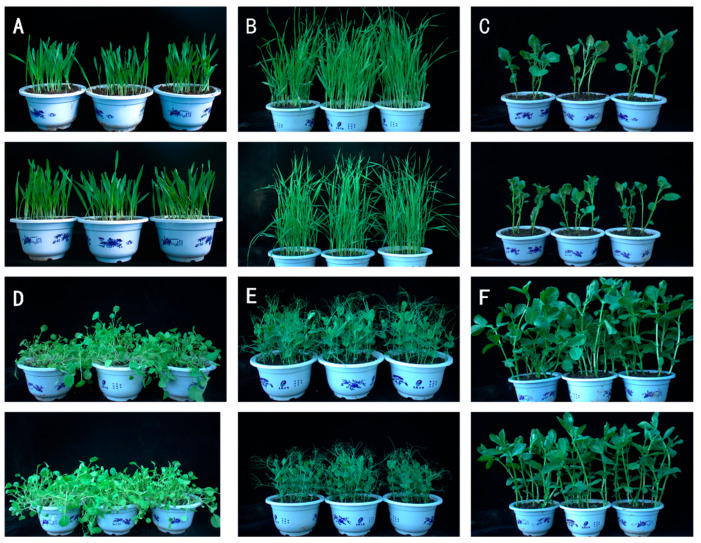
Effect of spore suspension of strain JZ-5 on crop safety. **Note**: (**A**) *Hordeum vulgare* var. *nudum* (control above, treatment below, with the same below); (**B**) *Triticum aestivum* L.; (**C**) *Solanum tuberosum* L.; (**D**) *Brassica napus* L.; (**E**) *Pisum sativum* L.; (**F**) *Vicia faba* L.

The spore suspension of strain JZ-5 was sprayed onto the test crops, and disease incidence was assessed 7 days post inoculation. The results are shown in Figure 3 and Table 3. Barley (*Hordeum vulgare* var. *nudum*), common wheat (*Triticum aestivum* L.), and potato (*Solanum tuberosum* L.) exhibited no symptoms in growth vigor or leaf color compared to the control group after 7 days of spraying. No disease spots were observed on the leaves, with both disease incidence and disease index recorded as 0, resulting in a safety classification of NS (Non-Susceptible). In contrast, rapeseed (*Brassica napus* L.) displayed leaf yellowing compared to the control, though no significant difference in growth was noted. The disease incidence and disease index were 3.33% and 0.67%, respectively, classifying it as LS (Low Susceptible). Pea (*Pisum sativum* L.) showed no visible symptoms, but exhibited stunted growth, with a disease incidence of 6.25% and a disease index of 1.25%, also categorized as LS. Faba bean (*Vicia faba* L.) developed leaf curling, along with scattered black lesions on the leaves. Its disease incidence and disease index reached 10% and 2%, respectively, and were similarly classified as LS.

### 3.4. Identification of the Strains

#### 3.4.1. Morphological Characterization of Strain JZ-5

**Figure 4:**
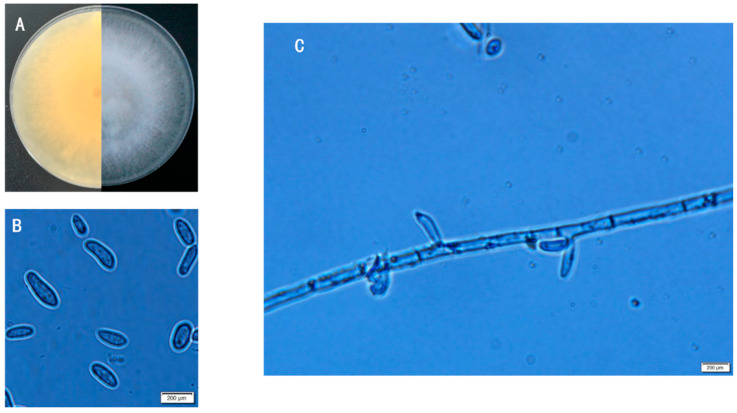
Morphological characteristics of strain ZJ-5. **Note**: (**A**) Colony; (**B**) Conidiophore; (**C**) Conidiophore. Scale bar: 200 μm.

The fungal strain JZ-5 exhibited distinct morphological characteristics when cultured on PDA medium. The colonies appeared as white fluffy mycelia during initial growth stages, developing a faint pink pigmentation on the reverse side during later phases (Figure 4A). The vegetative hyphae demonstrated vigorous growth with irregular, filamentous margins, lacking prominent elevation. Notably, the strain secreted a brownish-yellow pigment upon maturation. Microscopic examination revealed the presence of conidia developing intercalarily, among hyphal filaments. The spores displayed either ovoid or reniform morphology, appearing hyaline and aseptate under observation (Figure 4B,C).

#### 3.4.2. Molecular Characterization of *Fusarium oxysporum* Strain JZ-5

**Figure 5:**
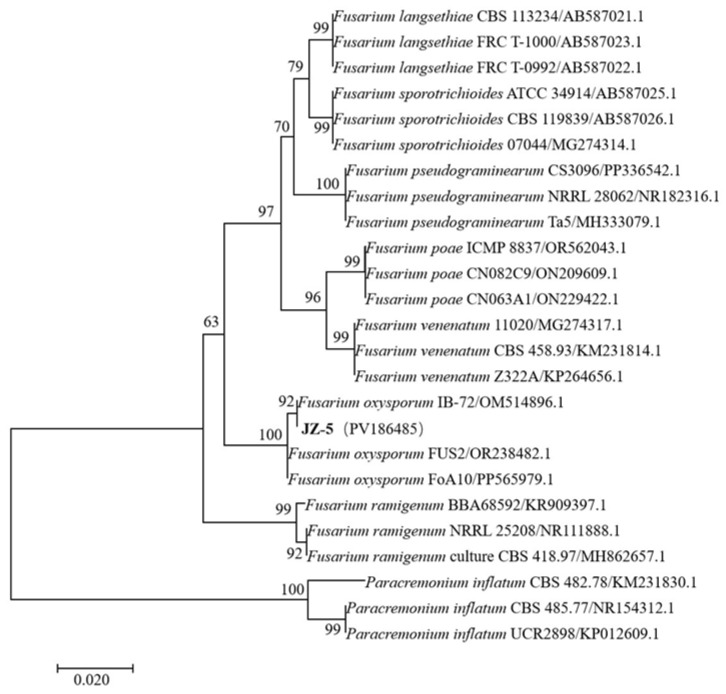
Phylogenetic tree of strain JZ-5 constructed based on the ITS gene sequences.

Strains were sequenced to select sequences to be saved in the NCBI database under accession number PV186485 (rDNA-ITS). Twenty-one sequences with high homology were selected, and three exogenous strains were used to construct the phylogenetic tree to make the developmental tree more convincing. The MEGA ClustalW algorithm was used for sequence comparison, and the Maximum Composite Likelihood method was used as the evolutionary model for phylogenetic analysis. The criteria for reconstructing the phylogeny were that, firstly, the DNA sequences were selected, and, secondly, the construction was carried out using the Maximum Composite Likelihood method, and replicates were validated by running 1000 bootstrap iterations. The rDNA-ITS sequence of JZ-5 was sequenced by BioCompany, and its length was 547 bp. The sequence is available in the NCBI database under accession number PV186485 (Figure 5) (rDNA-ITS). Twenty-one strains in the NCBI database with varying similarities were selected to construct a phylogenetic tree.,This analysis showed that strains with accession numbers OR238482.1 and pp565979.1 had 100% sequence similarity to *Fusarium oxysporum*, and OM514896.1 had 92% similarity, so the isolated strain JZ-5 was finally identified as *Fusarium oxysporum*, and named *Fusarium oxysporum* JZ-5.

#### 3.4.3. Observations on the Pathogenic Process of Strain JZ-05 on the Leaves of Winter Anemone Weeds

**Figure 6:**
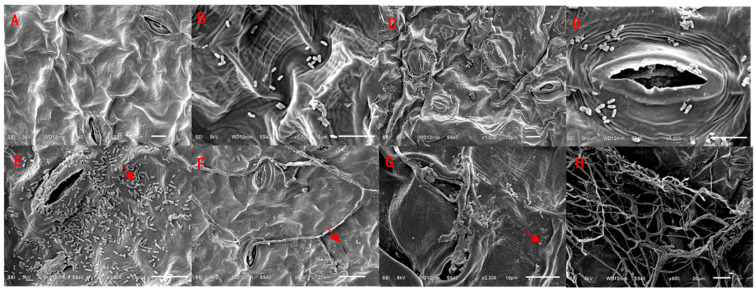
Scanning electron microscopic observation of tissue characteristics of *Malva verticillata* L. (Cluster mallow) infested by strain JZ-5 1–7d, the red arrows show the colonization of spores and hyphae on the leaf surface. **Note**: (**A**) Magnification: 1000×; Scale bar: 10 μm. (**B**) Magnification: 5000×; Scale bar: 5 μm. (**C**) Magnification: 1000×; Scale bar: 10 μm. (**D**) Magnification: 5000×; Scale bar: 5 μm. (**E**) Magnification: 2500×; Scale bar: 10 μm. (**F**) Magnification: 900×; Scale bar: 20 μm. (**G**) Magnification: 2300×; Scale bar: 10 μm. (**H**) Magnification: 600×; Scale bar: 20 μm.

Scanning electron microscopy observations revealed that the surface structure of uninoculated *Malva verticillata* L. (Cluster mallow) leaf tissue was intact (Figure 6A). At 1 d of inoculation, there were a small number of spores on the surface of the leaf tissue, no mycelium was found, and the structure of the leaf tissue was still intact (Figure 6B). At 2 d, there were a large number of spores on the leaf tissue surface, and the majority of the stomata were not open (Figure 6C). After 3–4 d, the spores became attached to the tissue surface, and were accompanied by mycelium invasion (Figure 6D,E). After 5–6 d, numerous spores and mycelia parasitized the surface of the tissue and absorbed nutrients (Figure 6F,G). After 7 d, the mycelium grew vigorously, and covered the surface of the tissue, so that the whole leaf tissue was damaged, which ultimately led to the formation of spots (Figure 6H).

## 4. Discussion

Modern agriculture predominantly relies on chemical herbicides for weed control, due to their superior short-term efficacy and cost-effectiveness compared to alternative weed management approaches [17]. However, these chemical agents exhibit significant environmental toxicity, frequently contaminating groundwater and accumulating within food chains through bioaccumulation. The persistent soil residues of certain herbicides have accelerated the emergence of resistant weed biotypes, consequently driving continuous increases in application rates and treatment frequencies [18,19]. This escalating resistance crisis has stimulated academic interest in establishing sustainable weed management systems, prompting extensive exploration of ecologically compatible alternative control strategies [20,21]. To reduce dependence on synthetic herbicides, biologically based weed control approaches utilizing bioactive components have gained increasing attention. Phytopathogenic organisms, particularly fungi, have emerged as prime targets for bioherbicide development, owing to their unique capacity for secondary metabolite biosynthesis [22]. Representing the most diverse and extensively utilized microbial group in bioherbicide applications, these fungi predominantly include the Fusarium, Alternaria, and Trichoderma genera [23]. Research demonstrates that bioactive compounds secreted by these microorganisms effectively penetrate plant cuticles, subsequently compromising membrane system integrity and disrupting cellular metabolic pathways, ultimately inducing characteristic necrotic lesions or systemic chlorosis in foliar tissues [24].

*Fusarium*, a genus of filamentous fungi capable of both plant parasitism and soil saprophytism, encompasses numerous phytopathogenic species. Upon infecting host plants, these pathogens trigger a series of physiological responses [25,26]. This study demonstrates the herbicidal potential of *Fusarium oxysporum* as a biocontrol agent against six weed species. Experimental results revealed strong pathogenicity toward *Elsholtzia densa Benth*., *Stellaria media* (L.) *Vill*., and *Lamium amplexicaule* L. Weed plants inoculated with a spore suspension (strain JZ-5) exhibited typical disease symptoms. Data indicated that the application of the spore suspension (1.0 × 10^6^ spores/mL) exhibited broad-spectrum herbicidal activity against common farmland weeds in the Qinghai Plateau region, with particularly high efficacy against *Lamium amplexicaule* L. (infection rate: 91.07%). The control efficiency exceeded 81% for both *Elsholtzia densa Benth*. and *Stellaria media* (L.) *Vill*. To our knowledge, this is the first report on the bioherbicidal potential of Fusarium against these weed species. Research indicates that Fusarium is the second most frequently cited fungal genus in mycotherbicide studies. Previous studies have confirmed that multiple Fusarium species—including *F. avenaceum*, *F. solani*, *F. acuminatum*, and *F. cerealis*—exhibit herbicidal activity against various Poaceae plants [27,28]. In our laboratory experiments, *Fusarium oxysporum* demonstrated significant weed suppression efficacy. Reliable species identification was achieved through ITS sequence analysis, traditional morphological classification, and phylogenetic analysis, which provided sufficient genetic evidence. Consequently, strain JZ-5 was taxonomically confirmed as *Fusarium oxysporum* [29].

Host specificity testing and environmental risk assessment are crucial for preventing potential harm to non-target plants and ecosystems caused by pathogenic fungi [30]. In this study, the *Fusarium oxysporum* strain JZ-5 demonstrated safety toward barley, wheat, and potato, while exhibiting mild pathogenicity toward oilseed rape and strong pathogenicity against faba bean and pea. These findings partially align with Cheng Haiyang’s report on the safety evaluation for oilseed rape and pea [31]. However, it should be noted that the current pathogenicity and crop safety assessments were conducted using only a single spore concentration. Subsequent studies employing gradient concentration experiments are required to establish the safe application threshold of this strain, thereby providing scientific guidance for field formulation development.

Current research has established a comprehensive consensus on the infection mechanisms of phytopathogenic fungi, which employ a multi-stage pathological process to colonize host plants. This process involves several critical pathological stages: specific adhesion and germination of conidia, appressorium differentiation, development of invasive hyphae, and subsequent tissue colonization [32]. During infection, fungal-secreted extracellular hydrolases (e.g., cellulases and pectinases) and secondary metabolites (including toxins and hormone analogs) synergistically disrupt the structural integrity of plant cell walls, ultimately leading to characteristic necrotic lesions or systemic chlorosis in leaf tissues [33]. Molecular pathological studies demonstrate that these bioactive compounds play pivotal roles in fungal pathogenicity by interfering with host-cell membrane permeability and metabolic homeostasis [34]. The works of He Yushan [35] and Xu Yueying [36] have validated the classical infection pattern of Fusarium species characterized by “initial stomatal invasion followed by progressive tissue degradation.” Furthermore, Lüth et al. [37] reported that Fusarium oxysporum excessively produces leucine, methionine, and tyrosine during infection, suggesting potential roles for these metabolites in fungal pathogenicity, though their precise mechanisms remain to be elucidated.

Scanning electron microscopy (SEM) observations further elucidated the infection dynamics of fungal pathogens on Malva verticillata L. leaves, revealing that fungal invasion primarily occurs through stomatal penetration. During 1–2 days post inoculation (dpi), hyphae were observed predominantly entering through stomatal openings. By 5–6 dpi, extensive colonization of spores and hyphae became evident on the leaf surface, accompanied by active nutrient absorption. At 7 dpi, vigorous hyphal proliferation completely covered the tissues, ultimately causing severe leaf damage. These findings demonstrate that fungal pathogens can effectively disrupt weed growth and development, corroborating previous studies which established that phytopathogenic fungi achieve weed control by suppressing weed growth and reproduction [38]. This study provides critical evidence for the development of microbial herbicides, as strain JZ-5 exhibits high pathogenicity against multiple weed species, including *Lamium amplexicaule* L., *Chenopodium album* L., and *Elsholtzia densa Benth*, indicating its potential application in agricultural weed management. Notably, this strain demonstrates broad-spectrum herbicidal activity while potentially posing lower environmental risks compared to conventional chemical herbicides, aligning with the principles of sustainable agriculture and eco-friendly practices. The SEM characterization has confirmed its stomatal invasion mechanism and clarified the infection process, thereby providing theoretical support for optimizing application strategies. Our laboratory is currently conducting further research on its formulation and evaluation as a bioherbicide agent.

## 5. Conclusions

In this study, strains isolated from diseased *Amaranthus retroflexus* L. samples served as test materials. Pot experiments were conducted using spore suspensions on six common farmland weeds in Qinghai Province. The results demonstrated significant pathogenic effects, with morbidity rates ranging from 15.28% to 91.07% among the tested weeds. Notably, *Lamium amplexicaule* L. exhibited the highest susceptibility, reaching a morbidity rate of 91.07%. Crop safety evaluations revealed that *Hordeum vulgare* var. *coeleste* L. (barley), *Triticum aestivum* L. (wheat), and *Solanum tuberosum* L. (potato) showed no adverse effects after 7 days of continuous inoculation with the fermentation broth. Strain identification was performed using morphological and molecular biological methods, including phylogenetic tree construction. Based on morphological characteristics and DNA sequence analysis, the strain was identified as *Fusarium oxysporum*, and designated *F. oxysporum* JZ-5.

## Figures and Tables

**Table 1:** Symptoms of strain JZ-5 infestation in four weed species.

Weed	Blade Area (cm^2^)	Incidence Area (cm^2^)	Incidence Grade	Incidence Characteristics
*Malva verticillata* L.	11.38 ± 1.30 a	11.08 ± 1.33 a	4	Leaf blade yellowing and softening, surface distribution of a large amount of mycelium, brownish and dead
*Polygonum lapathifolium* L.	7.84 ± 2.20 b	7.20 ± 2.53 b	4	Small brown spots around the fungus cake, leaf margins wrinkled and curled
*Polygonum aviculare* L.	1.79 ± 0.35 c	1.05 ± 0.13 c	2	Leaf discoloration and yellowing, mycelia cover the leaves
*Lamiuma mplexicaule* L.	3.16 ± 0.05 c	2.81 ± 0.61 c	4	Leaf blade turned completely yellow-brown, mycelium covers the whole leaf blade

**Note**: Data are the “mean ± standard error”. Different letters in the same column indicate significant differences (*p* < 0.05) by Duncan’s new complex polarity test.

**Table 2:** Symptoms of strain JZ-5 on the leaf area of six potted weed plants.

Weed	Morbidity Rate(%)	Disease Index	Incidence Grade
*Malva verticillata* L.	33.61 ± 3.75 b	8.36 ± 0.90 c	1
*Chenopodium album* L.	76.83 ± 1.78 a	38.36 ± 0.85 b	3
*Elsholtzia densa Benth*.	81.71 ± 1.66 a	61.26 ± 1.25 a	3
*Stellaria media* (L.) *Vill*.	75.17 ± 2.52 a	62.60 ± 10.90 a	3
*Polygonum lapathifolium* L.	23.46 ± 6.00 c	5.83 ± 1.52 c	1
*Lamium amplexicaule* L.	86.25 ± 5.43 a	64.63 ± 4.09 a	3

**Note**: Data are the “mean ± standard error”. Different letters in the same column indicate significant differences (*p* < 0.05) by Duncan’s new complex polarity test.

**Table 3:** Effects of spore suspension from strain JZ-5 on disease incidence and severity index in crops.

Crop	Morbidity Rate (%)	Disease Index (%)	Incidence Grade	Safety Grade
*Hordeum vulgare* var. *nudum*	0.00	0.00	0	NS
*Triticum aestivum* L.	0.00	0.00	0	NS
*Solanum tuberosum* L.	0.00	0.00	0	NS
*Brassica napus* L.	3.33	0.67	1	LS
*Pisum sativum* L.	6.25	1.25	1	LS
*Vicia faba* L.	10	2	1	LS

**Note**: **No Symptom (NS)**: Disease Index 0 ≤ DI < 5, indicating no adverse reactions observed. **Lightly Susceptible (LS)**: Disease Index 5 ≤ DI < 10, indicating mild and manageable adverse reactions present.

## Data Availability

The original contributions presented in this study are included in the article. Further inquiries can be directed to the corresponding author.
